# Sedimentation accelerates mixed-substrate conversion during sequential batch cultivation of a co-culture of sugar-specialist yeast strains

**DOI:** 10.1128/aem.00516-26

**Published:** 2026-07-16

**Authors:** Tobias Fecker, Michelle Rossouw, Jack T. Pronk, Rinke J. van Tatenhove-Pel

**Affiliations:** 1 Department of Biotechnology, Delft University of Technology 2860, Delft, the Netherlands; Chalmers tekniska hogskola AB, Gothenburg, Sweden

**Keywords:** sedimentation, yeast, mixed substrate, fermentation

## Abstract

Current industrial processes for yeast-based conversion of glucose, xylose, and arabinose mixtures in lignocellulosic hydrolysates mostly rely on monocultures of generalist strains that consume the sugars sequentially. Co-cultivation of “sugar-specialist” strains offers potential advantages regarding volumetric productivity and genetic stability of pentose-fermenting strains during prolonged operation. However, in these cultures, the slow fermentation of pentoses strongly affects the time required for complete sugar conversion. This study explores whether the kinetics of such co-cultures can be improved by increasing the biomass concentration through selective sedimentation of a slow-fermenting strain in sequential batch fermentations. Deletion of *ACE2* in a diploid, arabinose-specialist *Saccharomyces cerevisiae* strain yielded a multicellular, fast-sedimenting phenotype. In monocultures on 20 g∙L^−1^ arabinose in anaerobic sequential batch bioreactors with a 30-min biomass settling period between cultivation cycles, the sedimenting strain showed a shorter time for full conversion than its non-sedimenting parental strain (11.5 ± 0.4 h and 16.6 ± 0.4 h, respectively). Co-cultures of a glucose-specialist *S. cerevisiae* strain with the sedimenting arabinose specialist on a glucose-arabinose mixture (10 g·L^−1^ of each sugar) showed a 29% shorter batch time than corresponding co-cultures with the non-sedimenting arabinose specialist (12.6 ± 0.1 h vs. 17.7 ± 0.1 h). Increasing the biomass settling period to 1 h further reduced the batch time to 8.1 ± 0.1 h and resulted in near-simultaneous depletion of both sugars. These results show that if selective sedimentation of specific slow-fermenting strains can be implemented under relevant process conditions, it has the potential to improve microbial conversion of mixed substrates.

**IMPORTANCE:**

Fermentation of substrate mixtures from lignocellulosic biomass can reduce substrate costs but is often hindered by inefficient, sequential consumption of sugars by “sugar-generalist” *Saccharomyces cerevisiae* strains. While co-cultures of “sugar-specialist” *S. cerevisiae* strains can enable parallel consumption in sequential-batch fermentations, the overall volumetric productivity is limited by the slowest fermenter. This study presents a strategy to overcome this limitation by engineering a slow-fermenting arabinose-specialist *S. cerevisiae* strain for fast sedimentation and implementing a short settling phase between batch cycles in sequential-batch bioreactors, selectively concentrating the specialist’s biomass and improving consumption kinetics in subsequent cycles. This approach reduced overall fermentation time by 30% in monoculture and enabled near-simultaneous depletion of arabinose and glucose in co-culture with a glucose-specialist strain. These results show that selective sedimentation of co-culture partners can improve fermentation kinetics in sequential-batch cultures. If compatible with industrial process conditions, this strategy has the potential to improve the economics of co-culture-based fermentations.

## INTRODUCTION

Current large-scale processes in industrial biotechnology, including the production of ethanol with the yeast *Saccharomyces cerevisiae,* mainly rely on the conversion of “first-generation” feedstocks like hydrolyzed corn starch or cane sugar (1, 2). Over the past decades, the use of lignocellulosic biomass hydrolysates, derived from agricultural residues or energy crops, has received increased attention as a means to reduce land use and carbon emissions associated with ethanol production (1, 3). Unlike the glucose-rich hydrolysate obtained from corn starch or sugarcane, these second-generation feedstocks contain a mixture of the hexose glucose and the pentoses d-xylose and l-arabinose (henceforth xylose and arabinose) as major fermentable sugars (4).

To maximize productivity and product yield on the carbon source, all three sugars must be fermented rapidly and completely. However, native *S. cerevisiae* strains cannot ferment xylose and arabinose (3, 5). Intensive research efforts have successfully identified and engineered heterologous metabolic pathways that can be inserted into *S. cerevisiae*, enabling the organism to ferment all three lignocellulosic sugars in anaerobic batch cultures (4, 6). However, the fermentation kinetics of such "generalist" strains are often characterized by sequential sugar consumption (6, 7). These bi-phasic fermentation kinetics have been attributed to lower biomass-specific fermentation rates for the pentose sugars, glucose-repression mechanisms, and competitive inhibition of pentose uptake by glucose (5, 8). Delayed pentose consumption, which can be exacerbated by a diauxic adaptation phase, negatively affects volumetric productivity (9, 10).

An additional drawback of the use of generalist strains emerged in experiments with sequential batch cultures, in which each subsequent batch cultivation cycle is initiated with a culture sample from the previous cycle. In “single sugar” media, sequential batch cultivation can be used to increase volumetric productivity and even confers a selective advantage to fast-fermenting mutants (6, 11). In contrast, prolonged sequential batch cultivation of generalist strains on mixtures of glucose and pentose sugars resulted in a progressive increase in the time required for complete sugar conversion, caused by the deterioration of fermentation kinetics for both pentoses (7, 12).

To overcome the limitations of pathway degeneration and sequential consumption, previous studies constructed co-cultures of specialist *S. cerevisiae* strains, each consuming only a single sugar (7, 13). In these studies, a wild-type *S. cerevisiae* strain, able to consume only glucose, was cultured with other strains, engineered and evolved to only ferment the pentoses xylose or arabinose. Although this strategy prevented pathway degeneration and ensured parallel consumption of all sugars, it introduced new challenges related to the growth kinetics.

In a typical bioprocess, the consumption rate (*R_s_*, mmol∙L^−1^∙h^−1^) of a strain for a sugar is the product of its biomass concentration (C*_X_*, g∙L^−1^) and its biomass-specific consumption rate (*q_S_*, mmol∙g_Biomass_^−1^∙h^−1^) for this sugar (R_s_ = C_x_ ∙ q_s_) (14). Pentose-specialist strains often have a lower biomass-specific consumption rate than glucose specialists due to transport and regulation constraints, resulting in slow growth and consumption (R_S_) throughout the process (5, 15, 16). This results in biphasic growth dynamics, where the fast specialist consumes its sugar to depletion and then remains idle until the slower strains consume their designated sugar. On the other hand, because a generalist strain consumes the glucose first, it starts the pentose consumption phase with a higher biomass concentration (C_x_), augmenting subsequent pentose consumption rates and overall productivity (7). Increasing the initial inoculum size of the slower growing pentose specialists can mitigate this issue in batch cultures, but the solution is constrained by the total available cell mass for inoculation generated in the seed train (7, 17, 18).

Repeated or semi-continuous operation modes present an opportunity for optimizing the fermentation kinetics of sugar-specialist strains in consortia. These operation modes are characterized by the periodic removal of spent medium, often retaining the biomass, as is done extensively in the Brazilian bioethanol industry (19). Directly modifying relative inoculum sizes to enhance pentose fermentation rate is not possible in sequential batch co-cultures when each new cycle is inoculated with a representative sample of a previous, ideally mixed co-culture. However, one potential strategy to enhance the overall kinetics in such a system is to selectively increase the pentose specialists’ biomass concentration across the batch cycles through selective biomass retention—preferentially maintaining the biomass of the strain during a biomass-settling phase (14, 20). Selective retention would provide the slow-growing strain with a competitive advantage by starting the subsequent batch with a higher inoculum, thereby accelerating pentose consumption.

To achieve selective retention, the slow-growing strain must sediment more rapidly than the fast-growing strain in a co-culture. One way to selectively induce aggregation and sedimentation in *S. cerevisiae* cells involves deletion of the *ACE2* gene (21). *ACE2* encodes a transcriptional regulator that controls the expression of several genes involved in the final stage of the cell cycle, including the chitinase *CTS1* required for mother-daughter cell separation after budding (22). Inactivation of *ACE2* prevents separation of the budding cell from the mother, producing multicellular clonal aggregates (21, 23). Due to the different bud-localization preference in haploid and diploid *S. cerevisiae* cells, this effect is especially pronounced in diploid *ace2*∆ *ace2*∆ mutants, in which multicellular aggregates can reach diameters of hundreds of micrometers in size. These aggregates sediment rapidly and were previously shown to cause selective biomass retention in sequential batch cultures (21, 24).

In this study, we investigate whether selective sedimentation of the slowest growing strain in a consortium of sugar-specialist yeast strains can improve volumetric productivity during sequential batch bioreactor cultivation on sugar mixtures. We first engineered a diploid arabinose-specialist *S. cerevisiae* for rapid sedimentation by deleting both copies of *ACE2*. We then compared fermentation kinetics of this strain during growth in sequential batch fermentation on arabinose alone with those of the parental, non-sedimenting strain. Finally, we tested the impact of the sedimentation phenotype of the arabinose-specialist strain on fermentation kinetics in sequential batch cultures of a consortium with a glucose-specialist strain on a glucose-arabinose mixture.

## RESULTS

### Deletion of *ACE2* yields sedimenting, multicellular clusters in a diploid arabinose-consuming *S. cerevisiae* strains

*S. cerevisiae* IMS1278, obtained by a combination of target genetic modification and laboratory evolution, cannot consume glucose or xylose but was previously shown to consume arabinose in co-cultures with glucose- and xylose-specialist strains on a mixture of these three sugars (7). In this co-culture, the arabinose specialist consumed arabinose more slowly than the glucose strain consumed glucose (7). To mitigate the consumption difference in the co-culture, we wanted to construct and employ a sedimenting arabinose strain based on IMS1278.

**Fig 1:**
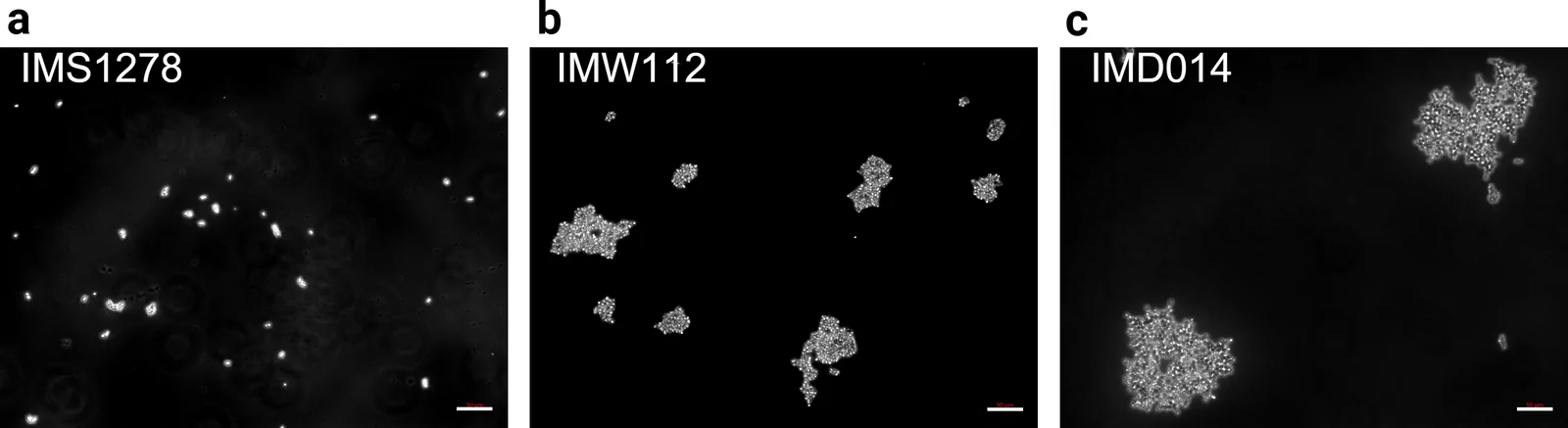
Occurrence of multicellular morphology in *S. cerevisiae* strains. (**a**) *S. cerevisiae* IMS1278; diploid *ACE2/ACE2* arabinose-specialist strain (see Table 2 for genotype description) grown in aerobic shake-flask culture on SM-arabinose (SMA). (**b**) *S. cerevisiae* IMW112; *∆ace2/∆ace2* mutant of strain IMS1278 grown in aerobic shake-flask culture on SM + arabinose (SMA). (**c**) *S. cerevisiae* IMD014; *∆ace2/∆ace2* pentose non-fermenting reference strain grown on SM + glucose (SMD). Samples were taken at stationary phase; marker bar indicates 50 µm.

We based our strategy to construct a fast-sedimenting arabinose-specialist strain on earlier work, which showed that inactivation of both copies of *ACE2* in diploid *S. cerevisiae* strains causes a multicellular, fast-sedimenting phenotype (21). Analysis of the genome sequence of IMS1278 with the MAGNOLYA algorithm (25) indicated that it had undergone whole-genome duplication (Fig. S1). We confirmed the diploidy by flow cytometry analysis of Sytox Green-stained suspensions, using *S. cerevisiae* strains with known ploidies as references (Fig. S2). Inactivation of both copies of *ACE2* in strain IMS1278 by Cas9-assisted genome editing yielded *S. cerevisiae* IMW112. In contrast to its parental strain IMS1278 (Fig. 1a), which showed a single-cell morphology, strain IMW112 formed multicellular clusters when grown in aerobic shake-flask cultures on synthetic medium with 20 g∙L^−1^ arabinose (SMA) (Fig. 1b). The diameter of these clusters was, however, smaller than those observed in cultures of the previously constructed, pentose-non-fermenting strain IMD014 grown on synthetic medium with 20 g∙L^−1^ glucose (SMD) (Fig. 1c) (21).

**Fig 2:**
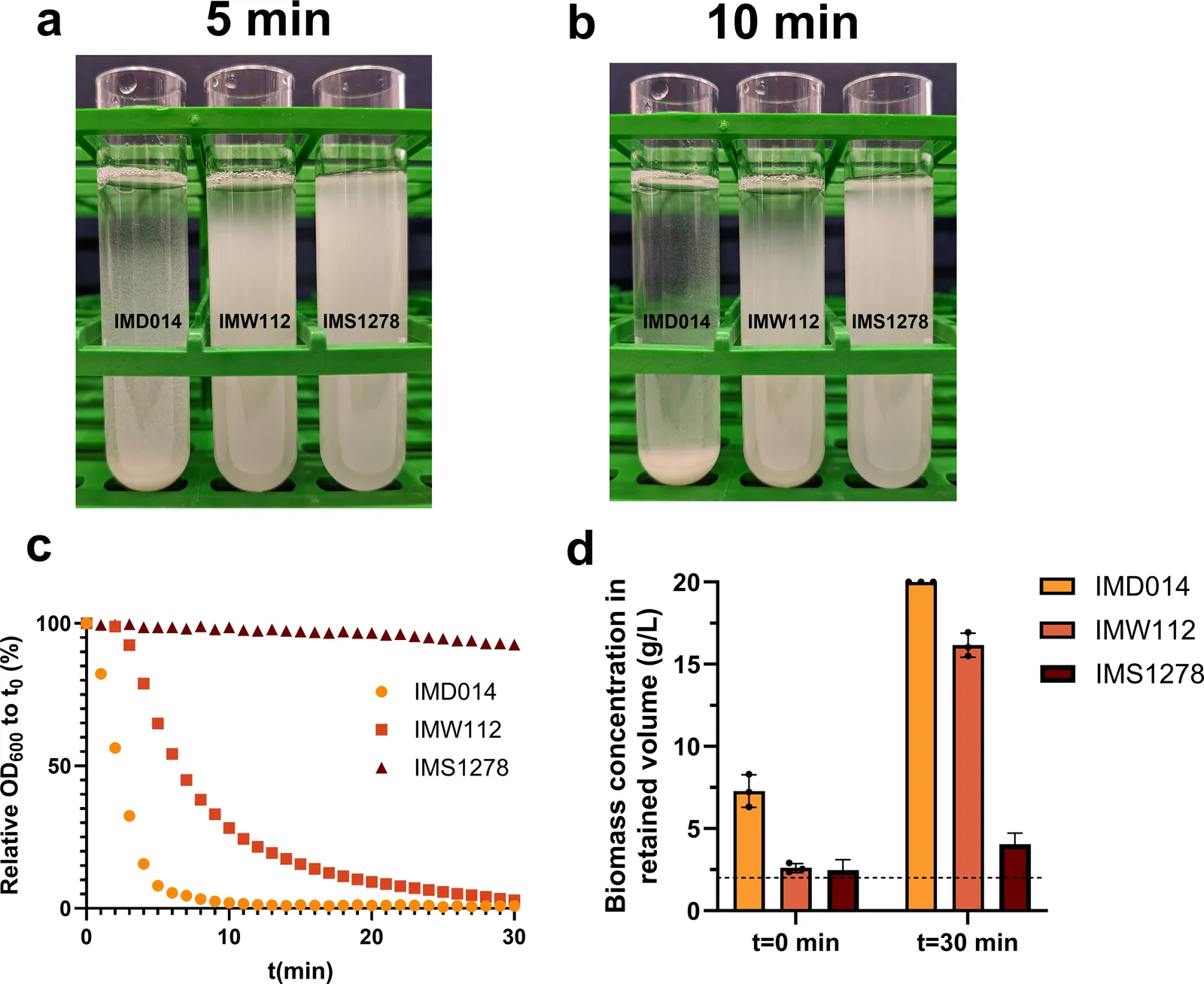
Sedimentation in suspensions of diploid *S. cerevisiae* strains *S. cerevisiae* IMD014 (*∆ace2/∆ace2* pentose non-fermenting reference strain), IMW112 (*∆ace2/∆ace2* mutant of arabinose specialist strain IMS1278; see Table 2 for genotype description), and IMS1278 (*ACE2/ACE2* arabinose-specialist strain). (**a**) Premixed cell suspensions after 5 min of static incubation. (**b**) Premixed cell suspensions after 10 min of static incubation. (**c**) Monitoring of optical density at 660 nm (OD660) during static incubation of cell suspensions in a spectrophotometer. (**d**) Simulation in 15 mL glass tubes of biomass sedimentation during the 10-min emptying phase of a sequential batch bioreactor. After adding 10 mL of a premixed 2.0 g biomass∙L^*−1*^ cell suspension, 1 mL of the samples was removed at 1-min intervals from a point corresponding with a residual volume of 1 mL. The biomass concentration in the remaining 1 mL suspension was then measured. Experiments were performed with and without a prior 30 min settling period. Data represent average ± SD of three replicate experiments for each strain. Dotted line represents the starting concentration of 2.0 g biomass∙L^−1^. Strains were pre-grown as indicated in the legend of Fig. 1.

To test whether the multicellular morphology of strain IMW112 led to fast sedimentation, briefly vortexed cell suspensions of strains IMW112, IMS1278, and IMD014 were incubated in stationary glass tubes. After 5 and 10 min, clear zones appeared at the top of suspensions of each of the two *∆ace2/∆ace2* strains, while no such zones were observed in suspensions of the *ACE2/ACE2* strain IMS1278 (Fig. 2a and b). These results showed that consistent with its smaller cell cluster size (Fig. 1), strain IMW112 sedimented slower than the pentose non-fermenting *∆ace2/∆ace2* strain. A further comparison of the sedimentation dynamics of these three strains confirmed this interpretation. When premixed cell suspensions were statically incubated in a spectrophotometer cuvette, strain IMW112 reached 25% of the initial OD_660_ after 10 min, while strain IMD014 already reached this reduction after 5 min (Fig. 2c).

To investigate if the sedimentation rate of strain IMW112 would be sufficient to support cell retention in a sequential-batch bioreactor setup with a 10-min emptying phase, a simulation experiment was performed in 15 mL tubes. We added 10 mL culture to the tube and removed 1 mL from the 1 mL (10% volume) mark at 1-min intervals, which corresponds to the medium removal in a bioreactor. Subsequently, we assessed the biomass concentration in the remaining 1 mL by measuring the biomass in the removed fraction and subtracting it from the total biomass. When no prior static incubation step was performed, the biomass in the remaining suspension had increased by 3.5-fold for the IMD014 strain, but only by 1.3-fold in the IMW112 strain and remained at the initial concentration in the *ACE2*/*ACE2* strain IMS1278 (Fig. 2d). After implementing a 30-min static incubation phase prior to initiating the emptying steps, the residual biomass concentration in the remaining 1-mL sample of suspensions of strain IMW112 was 8-fold higher than the initial biomass concentration. When subjected to the same procedure, strain IMS1278 showed an approximately 2-fold higher biomass concentration in the final sample (Fig. 2d). Based on these observations, a settling time of 30 min was chosen for sequential batch bioreactor experiments.

### Biomass retention and arabinose conversion kinetics in sequential-batch monocultures

**Fig 3:**
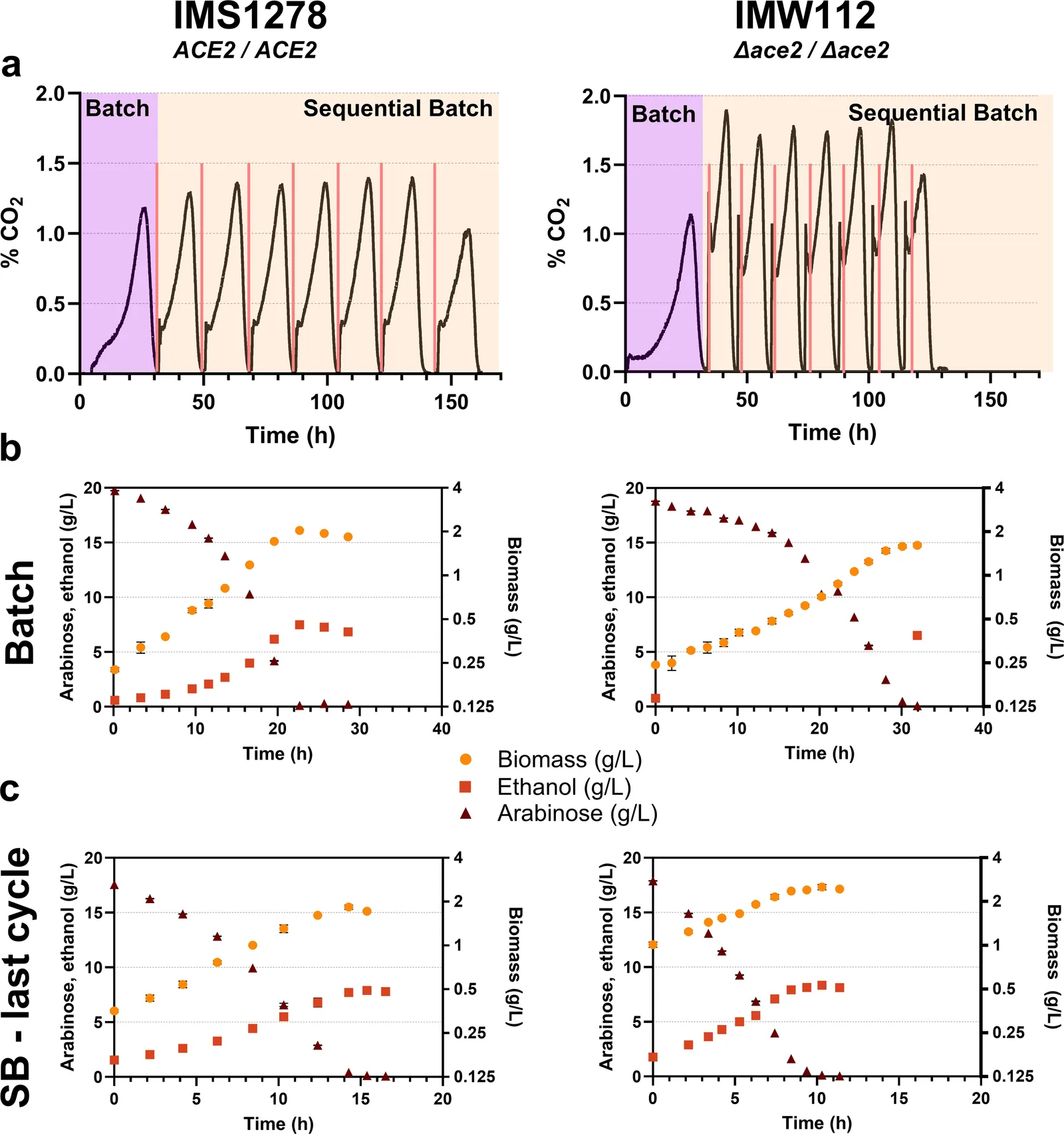
Fermentation kinetics of diploid *S. cerevisiae* strains IMS1278 (*ACE2/ACE2* arabinose-specialist strain, left-hand panels; see Table 2 for genotype description) and IMW112 (*∆ace2/∆ace2* mutant of strain IMS1278, right-hand panel), grown in anaerobic sequential-batch bioreactors on SM with 20 g · L^*−1*^ arabinose. (**a**) CO_2_ content of bioreactor off-gas during the initial batch culture and seven subsequent sequential batch cycles. Off-gas data for the final cycle are influenced by sampling for metabolite analyses. The 30-min settling phase is indicated in red. (**b**) Concentrations of arabinose, biomass, and ethanol in the initial anaerobic batch cultures of each strain. Cultures were inoculated with 0.2 g∙L^−1^ biomass. Due to an issue with the ethanol sampling procedure, only start- and end-point ethanol concentrations could be determined for the IMW112 strain. (**c**) Concentrations of arabinose, biomass, and ethanol in the seventh cycle of sequential-batch operation of each strain. Metabolite and biomass data shown in the figure represent average ± SD of technical replicates. Duplicate bioreactors were run for each strain, with data from one reactor shown in the figure; data for the other reactors are shown in Fig. S3.

To compare biomass retention and arabinose conversion kinetics of the arabinose-specialist strains IMS1278 (*ACE2/ACE2*) and IMW112 (*∆ace2/∆ace2*) in anaerobic sequential batch cultures on SMA, they were first grown as monocultures. In the initial bioreactor batch cultures, strain IMW112 grew more slowly than strain IMS1278 (Fig. 3a and b, IMW112: µ_max_= 0.080 h^−1^; µ_max_= 0.099 h^−1^, respectively, Table 1). The time required to achieve full arabinose depletion (pCO_2_ in off-gas <0.05%) for strain IMS1278 was 24.1 ± 1.6 h, as compared to 31.5 ± 0.5 h for the strain IMW112 (Fig. 3b). The ethanol yield was similar for both strains (Table 1).

**TABLE 1:** Maximum specific growth rate (µ_max_), biomass yield, and ethanol yield of diploid *S. cerevisiae* strains IMS1278 and IMW112^*a*^

Strain and tested cycle	µ_max_ (h^−1^)	Biomass yield (g_x_/g_s_)	Ethanol yield (g_etoh_/g_s_)
IMS1278 batch	0.099 ± 0.008	0.088 ± 0.002	0.36 ± 0.01
IMS1278 SBR last cycle	0.14 ± 0.01	0.083 ± 0.000	0.36 ± 0.00
IMW112 batch	0.080 ± 0.002	0.078 ± 0.003	0.35 ± 0.00
IMW112 SBR last cycle	0.10 ± 0.000	0.082 ± 0.000	0.36 ± 0.00

^*a*^ IMS1278 is an *ACE2/ACE2* arabinose-specialist strain (see Table 2 for genotype description), and IMW112 is an *∆ace2/∆ace2* mutant of strain IMS1278. Strains were grown in anaerobic sequential batch bioreactors on SM with 20 g · L^*−*1^ arabinose. Data are presented for initial anaerobic batch cultures and the last of seven subsequent sequential-batch cycles of each strain. Data represent average ± SD of duplicate reactors for each strain.

To investigate the impact of biomass retention of the sedimenting *∆ace2/∆ace2* strain IMW112 on fermentation kinetics in sequential-batch cultures, we compared its performance in such cultures with that of the non-sedimenting *ACE2/ACE2* strain IMS1278. The sequential-batch bioreactors were programmed to automatically switch off the stirrer and aeration when a decrease in the CO_2_ concentration below a preset value indicated arabinose depletion. After a 30-min settling time, 90% of the culture volume was then automatically replaced by fresh medium (see methods section).

Except for a difference in the specific growth rate, the two strains showed similar off-gas CO_2_ profiles in the batch culture that preceded the sequential-batch mode (Fig. 3a). After this, the non-clustering *ACE2/ACE2* strain IMS1278 maintained the same profile in each subsequent cycle, and the *∆ace2/∆ace2* strain IMW112 started each subsequent cycle with a higher initial off-gas CO_2_ concentration, which was indicative of biomass retention (Fig. 3a). In addition, during sequential-batch operation with strain IMW112, the duration of each cycle was shorter than the length of the initial batch phase (Fig. 3a). Biomass and metabolite analyses in the seventh cycle of sequential-batch operation confirmed the impact of sedimentation. Reactors with strain IMW112 initiated this cycle with 0.97 ± 0.06 g∙L^−1^ biomass, resulting in arabinose depletion (pCO_2_ in off-gas <0.05%) in 11.3 ± 0.4 h. This time was 32% shorter than observed in reactors with strain IMS1278, which started at 0.33 ± 0.03 g∙L^−1^ biomass and required 16.6 ± 0.4 h for arabinose depletion (Fig. 3c). Both strains show a higher specific growth rate in the seventh cycle of sequential-batch operation than in the initial anaerobic batch cultures, which may indicate an adaptation to the anaerobic conditions (Table 1).

The results show that accelerated sedimentation of strain IMW112 resulted in biomass retention in sequential batch cultures, thereby enabling higher volumetric conversion rates than observed with a reference strain.

### Selective sedimentation of an arabinose-specialist strain accelerates the conversion of a glucose-arabinose mixture in sequential-batch co-cultures

After demonstrating that sedimentation of the clustering strain IMW112 resulted in faster arabinose consumption after multiple sequential batch cycles, we next investigated the impact of selective sedimentation on the kinetics of mixed-substrate conversion. We characterized the first and last cycle of an anaerobic sequential batch setup with 10 g∙L^−1^ each of arabinose and glucose, inoculated with 0.1 g∙L^−1^ CEN.PK113-7D and 0.1 g∙L^−1^ of either the non-clustering IMS1278 strain or the clustering IMW112 strain. By halving both the initial concentration of arabinose and the inoculation size of the arabinose specialist, we maintained the same biomass-to-substrate ratio as in the monoculture experiments. Therefore, we expected the time required for arabinose depletion to be similar to that observed for the monocultures.

**Fig 4:**
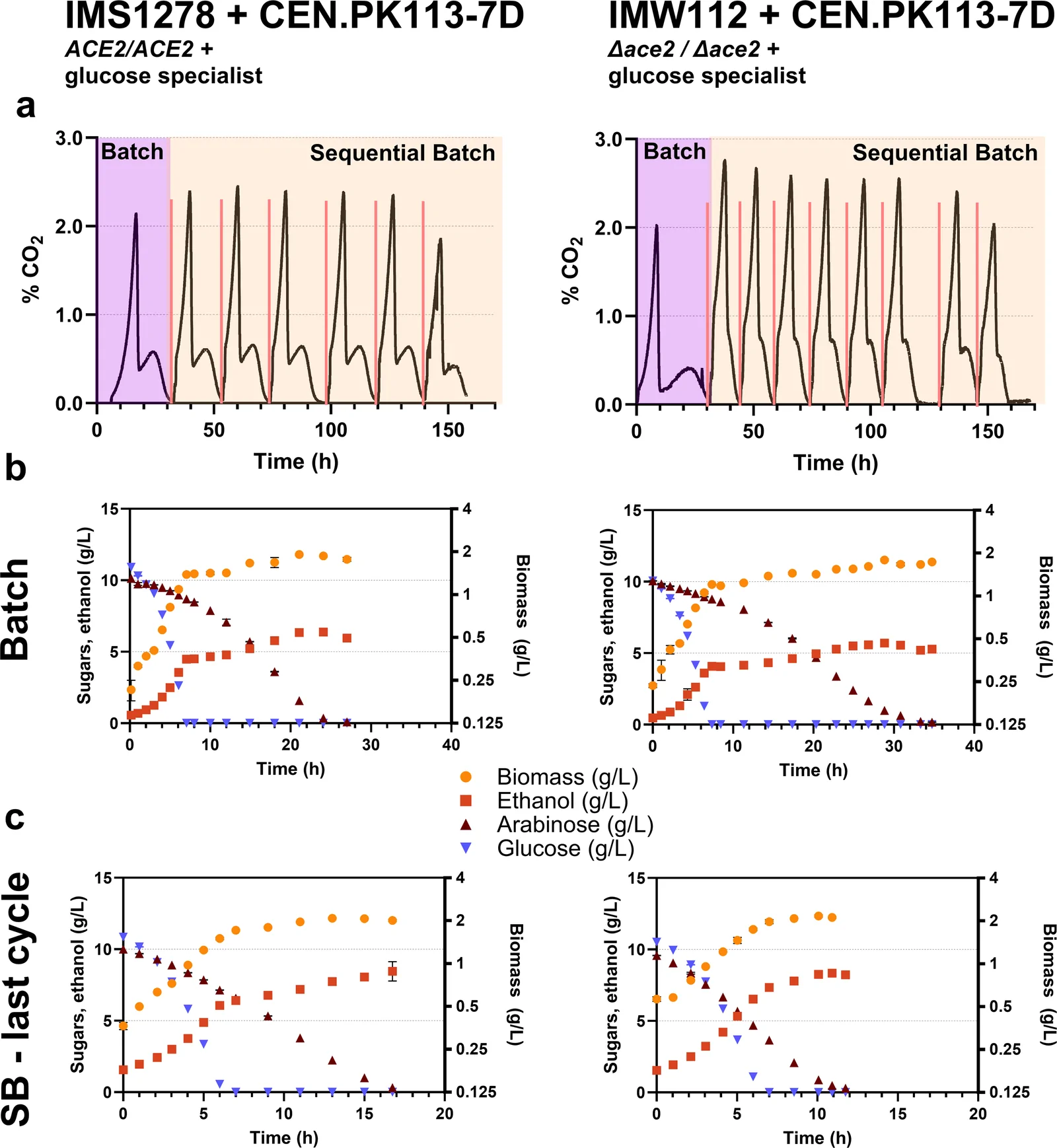
Fermentation kinetics of co-cultures of the pentose non-fermenting reference strain (“glucose specialist”) *S. cerevisiae* CEN.PK with either strain IMS1278 (*ACE2/ACE2* arabinose-specialist strain, left-hand panels; see Table 2 for genotype description) or IMW112 (*∆ace2/∆ace2* mutant of strain IMS1278, right-hand panels), grown in anaerobic sequential-batch bioreactors on SM with 10 g · L^−1^ each of glucose and arabinose. (**a**) CO_2_ content of bioreactor off-gas during the initial batch culture and subsequent sequential-batch cycles. Off-gas data for the final cycles are influenced by sampling for metabolite analyses. The 30-min settling phase is indicated in red. (**b**) Concentrations of arabinose, glucose, biomass, and ethanol in the initial anaerobic batch cultures of each co-culture, inoculated with 0.1 g biomass∙L^−1^ of each strain. (**c**) Concentrations of arabinose, glucose, biomass, and ethanol in the final cycle of sequential-batch operation. Metabolite and biomass data shown in the figure represent average ± SD of technical replicates. Duplicate bioreactors were run for each strain combination, with data from one reactor shown in the figure; data for the other reactors are shown in Fig. S4.

The off-gas CO_2_ profile for the batch phase was similar for both strain combinations and showed two distinct peaks, suggesting two growth phases in the culture (Fig. 4a). The biphasic CO_2_ profiles were consistent with complete glucose consumption, in both co-cultures, within 7.5 h ± 0.2 h. Complete conversion of arabinose occurred within 24.8 ± 0.7 h and 30.1 ± 0.1 h for strains IMS1278 and IMW112, respectively (Fig. 4b). Since the time to full conversion of arabinose was similar in co-culture as in monoculture, this suggests that the strains were unaffected by the presence of the glucose specialist. The faster consumption of arabinose by strain IMS1278 was consistent with its faster growth in anaerobic monocultures on arabinose (Fig. 3).

After the initial batch phase, the co-cultures were subjected to the same sequential-batch regime as described above for monocultures of the arabinose-specialist strains. Through six sequential-batch cycles, co-cultures of strain CEN.PK113-7D with the arabinose-specialist strain IMS1278 (*ACE2/ACE2*) maintained a CO_2_ off-gas profile with two distinct peaks, while these distinct peaks were nearly absent in the co-culture with IMW112 (*∆ace2/∆ace2*) after the first cycle (Fig. 4a). Analysis of the final cycle of these sequential batch cultures showed initial biomass concentrations of 0.34 ± 0.03 g∙L^−1^ and 0.56 ± 0.00 g∙L^−1^ for co-cultures with IMS1278 and IMW112, respectively (Fig. 4c). In all four final cultures, glucose was consumed within 7.8 ± 0.3 h. In these final cycles, co-cultures with strain IMW112 converted arabinose faster than strain IMS1278 (12.6 ± 0.1 h and 17.7 ± 0.1 h required for arabinose conversion, respectively). This reduction by 29% is consistent with the results obtained for the monocultures.

**Fig 5:**
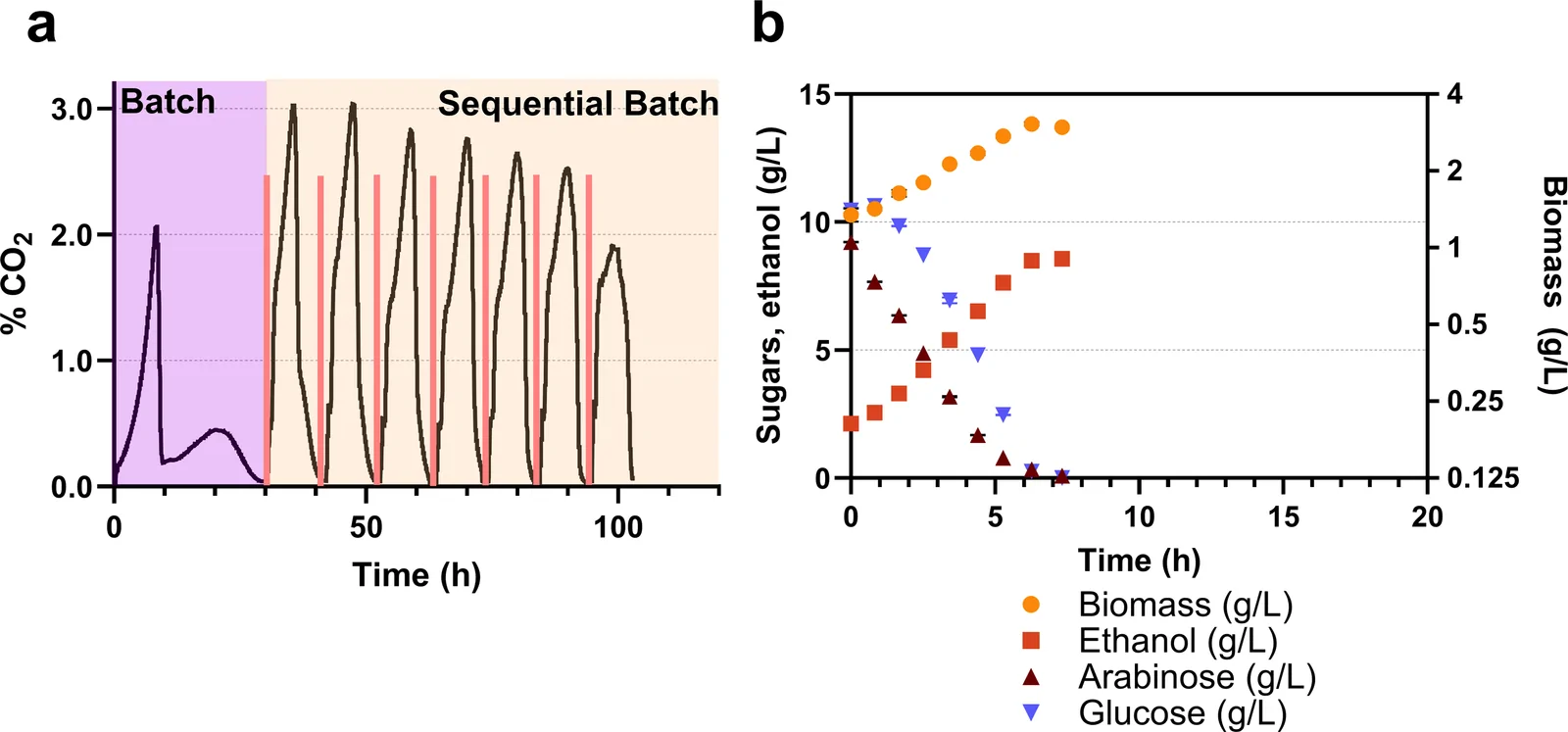
Fermentation kinetics of co-cultures of the pentose non-fermenting reference strain (“glucose specialist”) *S. cerevisiae* CEN.PK and the arabinose-specialist strain IMW112 (*∆ace2/∆ace2*) grown in anaerobic sequential-batch bioreactors on SM with 10 g · L^−1^ each of glucose and arabinose. In comparison with the experiments shown in Fig. 4, settling time after substrate depletion was extended from 30 to 60 min. (**a**) CO_2_ content of bioreactor off-gas during an initial batch culture and subsequent sequential-batch cycles. Off-gas data for the final cycle are influenced by sampling for metabolite analyses. The 60-min settling phase is indicated in red. (**b**) Concentrations of arabinose, glucose, biomass, and ethanol in the final cycle of sequential-batch operation. Metabolite and biomass data shown in the figure represent average ± SD of technical replicates. Duplicate bioreactors were run, with data from one reactor shown in the figure; data for the other reactor are shown in Fig. S5.

To investigate if the positive impact of selective sedimentation of the arabinose-specialist strain IMW112 on arabinose conversion kinetics in sequential-batch co-cultures with the glucose specialist strain CEN.PK could be further increased, the settling time was increased from 30 min to 60 min. During sequential-batch cultivation, the off-gas CO_2_ profile of these co-cultures showed only a single peak as opposed to the two distinct peaks in the first cycle, suggesting that both sugars were consumed simultaneously (Fig. 5a). Measurements on the seventh cycle of duplicate cultures showed that the initial biomass concentration had increased to 1.36 ± 0.02 g∙L^−1^, allowing the co-culture to simultaneously consume both sugars within 8.1 ± 0.1 h (Fig. 5b). These results show that the consumption rate of the sedimenting strain can be adjusted by changing the biomass settling time.

## DISCUSSION

In this study, we explored the application of sedimenting-specialist *S. cerevisiae* strains to intensify mixed-substrate processes. Deletion of *ACE2* caused the strains to form multicellular clusters that settled more quickly than their unicellular progenitors. Increasing the relative biomass of the slower strain during repeated batch fermentation enabled fast and parallel consumption of all available carbon sources.

As previously shown in wild-type *S. cerevisiae* strain backgrounds (21, 26), inactivation of *ACE2* in an engineered, diploid arabinose-specialist strain of *S. cerevisiae* conferred a multicellular, sedimenting phenotype (Fig. 1 and 2). In anaerobic, arabinose-grown monocultures, the specific growth rate of the resulting strain was approximately 20% lower than that of its *ACE2/ACE2* parental strain (Table 1). The combination of a small size and low specific growth rate of the multicellular clusters (Fig. 1), combined with the high (20 g · L^−1^) arabinose concentrations used in these cultures, rules out diffusion limitation as a plausible explanation for this difference in growth rate. Instead, slower growth of the cluster-forming strain may reflect steric hindrance and/or mechanical stress experienced by cells in the clusters’ cores (27). Similarly, the small cluster size would likely not present a barrier for oxygen transfer to the center cells under aerobic conditions.

Per Stokes’ law, the sedimentation velocity of a particle is proportional to the square of the cluster diameter. Arabinose-grown anaerobic cultures of the *∆ace2/∆ace2* arabinose-specialist strain showed smaller clusters and, consequently, slower sedimentation than glucose-grown cultures of a congenic *∆ace2/∆ace2* reference strain (Fig. 1 and 2). In clonal, multicellular clusters, size is determined by cluster age and its internal biophysical properties, particularly the maximal internal stress the cluster can withstand before fracture (28, 29). Previous studies have shown that the morphology of the constituent cells governs the cluster’s stress tolerance (23, 28, 30). The observed differences in cluster size between the two strains may therefore be caused by differences in cell size and aspect ratio (31). Alternatively, it might reflect different residual expression levels of *CTS1*, which encodes the endochitinase involved in mother-daughter cell separation, in the absence of its regulator Ace2 (32). Cell cluster size and settling rate of the sedimenting strain may be increased via evolutionary methods (33).

In anaerobic, arabinose-grown sequential-batch bioreactors, with a sedimentation time of 30 min between cycles, monocultures of the *∆ace2 ∆ace2* arabinose-specialist showed clear biomass retention. This enabled shorter cycle times than in similar cultures of its congenic, non-sedimenting parental strain (Fig. 3). Use of biomass sedimentation to enhance volumetric conversion rates in sequential batch cultures is, in itself, not a new concept. Granular-sludge systems are applied on a large scale in microbial wastewater treatment (34), flocculation is used for collection and subsequent repatching in brewing (35), and large-scale Brazilian bioethanol processes achieve up to 600 fermentation cycles per harvest season through the use of centrifugation to remove product and recycle biomass (14, 19). For the latter process, sedimentation via flocculation has been suggested as a cost-saving alternative for the centrifugation step (36). Bioreactor designs enabling the continuous removal of sedimenting cells in a binary culture have been explored previously and may be integrated in processes at larger scale (37). We are not aware of prior studies that explore selective sedimentation of co-culture partners to balance their relative contributions to mixed-substrate conversion.

The present study shows that in sequential-batch co-cultures of a fast-fermenting glucose-specialist strain and a much slower fermenting arabinose-specialist strain, cycle times for the conversion of a glucose-arabinose mixture were strongly reduced by selective sedimentation of the latter during a short settling phase (Fig. 4 and 5). This work may be extended to also include a xylose-specialist yeast strain. If this concept can be applied for mixed-substrate conversion in large-scale processes, its advantages may extend beyond volumetric productivity. When, in larger cell clusters, specific growth rates can be significantly reduced, this may enhance yields on substrates of dissimilatory products such as ethanol by increasing the relative contribution of maintenance energy metabolism to overall conversion rates (38, 39). In addition, yeast-cell aggregates formed by flocculation have been shown to exhibit increased tolerance to inhibitors occurring in lignocellulosic hydrolysates (40).

We realize that the defined media and laboratory conditions employed in our study do not capture key challenges associated with feedstocks and bioreactor scales used in industry. Viscosity of the slurries obtained after hydrolysis of lignocellulosic feedstock is a known challenge for gravity-based biomass recycling, often addressed by integrating a liquid-solid separation step to lower viscosity (41, 42). Application in such systems is likely to require further metabolic and/or evolutionary engineering to enhance biomass settling (33). The simple approach described here may, however, be of immediate relevance in other co-culture contexts. For example, in division-of-labor strategies that distribute enzymes of long biosynthetic pathways for complex biomass components (43), co-culture partners that carry pathway enzymes with low k_cat_ values may be specifically retained in sequential-batch cultures to maximize volumetric productivity.

This study was primarily inspired by industrial application of co-cultures. However, its results also illustrate how simple mutations that result in the formation of multicellular clusters can confer a selective benefit to slow-growing microbial species and communities in micro-environments that naturally undergo “overflow” events (44). For yeasts such as *S. cerevisiae*, whose natural environments are associated with plants and trees (45), these might include environments subject to periodic washout by rainfall (e.g., tree bark or flower petals). Investigating if a sedimenting phenotype contributes to persistence provides an interesting avenue for ecological research.

## MATERIALS AND METHODS

### Microbial strains and growth conditions

The *S. cerevisiae* strains used in this study (Table 2) were derived from the CEN.PK lineage, except for FRY153 (46, 47). Synthetic medium (SM), containing 5.0 g∙L^−1^ (NH_4_)_2_SO_4_, 3.0 g∙L^−1^ KH_2_PO_4_, 0.5 g∙L^−1^ MgSO_4_⋅7H_2_O, trace elements, and vitamins, was prepared as described previously (48). Freezer stocks were prepared by growing the strains in SM with 20 g∙L^−1^ l-arabinose and storing the samples from exponentially growing cultures at −80˚C with 30% (vol%) glycerol. Yeast strains were pre-cultured in 100 mL SM with 20 g∙L^−1^ of the appropriate sugar in round-bottom 500 mL shake flasks in a shaking incubator at 30˚C. Anaerobic growth medium was supplemented with ergosterol (10 mg∙L^−1^) and Tween80 (420 mg∙L^−1^) (49). Chemically competent *Escherichia coli* XL1-Blue cells were cultured on Lysogeny Broth, supplemented with 100 mg∙L^−1^ ampicillin (Merck, Darmstadt, Germany) where relevant. Solid media were prepared by adding 20 g∙L^−1^ agar (Becton Dickinson, Franklin Lakes, NJ) prior to heat sterilization. *E. coli* cultures on agar plates were incubated overnight at 37˚C, and *S. cerevisiae* plates were incubated at 30˚C for 3 days.

**TABLE 2:** *S. cerevisiae* strains used in this study

Strain name	Relevant genotype	Description	Reference
*S. cerevisiae* CEN.PK113-7D	*MATa MAL2-8c SUC2*	Common laboratory strain (wild-type, “glucose-specialist strain”)	(46, 47)
*S. cerevisiae* IMS1278	*MATa, ura3-52, his3-1, leu2-3112, MAL2-8c, SUC2, Δglk1::*Sp*HIS5, Δhxk1::*Kl*LEU2, Δgal1::CAS9- AMDS, Δgre3::*(pTDH3*_RPE1,* pPGK1*_TKL1_*tTKL1*,* pTEF1*_TAL1_*tTAL1*,* pPGI1*_NQM1_*tNQM1*,* pTPI1*_RKI1*_tRKI1*,* pPYK1*_TKL2_*tTKL2)*, Δgal80::* (pTPI*_*La*araA_*tCYC)∗*9,* pPYK*_*La*araB_*tPGI1*,* pPGK*_*La*araD_*tTDH3*, Δhxk2::*Pc*ARAT*; genetic changes that occurred during evolution: whole-genome diploidization, *GAL2* (N376I T89I), *BMH1* (E22*)*, IPT1* (S313F)	Diploid arabinose-specialist strain that has undergone laboratory evolution on a mixture of arabinose, glucose, and xylose under anaerobic conditions first in monoculture, and then in co-culture with a glucose- and a xylose-specialist strain; all modifications are homozygous	(7, 50)
*S. cerevisiae* IMW112	IMS1278 *Δace2/Δace2*	Cluster-forming, fast-sedimenting diploid arabinose specialist strain generated by deleting both copies of *ACE2*	This work
*S. cerevisiae* IMD014	*MAT*a/α *ura*3*-52*/*URA*3 *ace*2-2-loxP-*HphNT1*-loxP/*ace*2-2-loxP-*KanMX*-loxP	Diploid, cluster-forming, fast-sedimenting glucose specialist generated by mutating *ACE2* in both alleles	(21)
*S. cerevisiae* CEN.PK122	*MATa/MATα*	Laboratory strain, diploid	(46, 47)
*S. cerevisiae* FRY153	3n	Triploid strain	(51)

### Plasmid construction

A Cas9 gRNA target sequence in *ACE2* was identified as described previously (52). To construct plasmid pUDR913 (Table S1) containing the gRNA, a linear backbone fragment of pROS13 was first PCR-amplified with primer 6005 (Table S2), and the insert containing the gRNA was PCR-amplified with primer 20864 using Phusion DNA polymerase (Thermo Fisher). Backbone and insert were isolated from the agarose gel using the Zymoclean Gel DNA Recovery Kit (Zymo Research, Irvine, CA). The plasmid was assembled using Gibson Assembly using a HiFi DNA Assembly master mix (New England Biolabs, Ipswich, MA). One microliter of the mixture was used to transform *E. coli* XL1-Blue cells with a heat-shock protocol (53). Plasmid pUDR913 was isolated from the transformed *E. coli* cells using the Sigma GenElute Plasmid Miniprep Kit (Sigma-Aldrich).

### Gene editing

A dsDNA repair fragment for deletion of *ACE2* was prepared by mixing oligonucleotides 20865 and 20866 (Table S2) in a 1:1 molar ratio. The mixture was heated to 95˚C for 5 min and cooled on ice. *S. cerevisiae* IMW112 was constructed by co-transforming strain IMS1278 with gRNA-plasmid pUDR913 (Table S1) and the *ACE2* repair fragment, using the lithium-acetate method (54). Transformants were selected on SM plates supplemented with 20 g∙L^−1^ arabinose and 200 µg∙L^−1^ hygromycin B. Correct deletion was checked by diagnostic PCR with DreamTaq polymerase (Thermo Fisher) using primers 20867 and 20868 (Table S2). pUDR913 was removed by growing transformants with confirmed genotype on non-selective SM supplemented with 20 g∙L^−1^ arabinose, after which a cured IMW112 strain was stored at −80˚C.

### Ploidy analysis using flow cytometry

The ploidy of *S. cerevisiae* IMS1278 was determined using a flow cytometry protocol adapted from previously published work (55). Cells were grown aerobically in SM + 20 g∙L^−1^ arabinose for 24 h. In parallel, *S. cerevisiae* haploid, diploid, and triploid (strains CEN.PK113-7D, CEN.PK122, and FRY153, respectively, Table 1) were grown overnight in SM + 20 g∙L^−1^ glucose. After harvesting approximately 10^7^ cells and washing once with demineralized H_2_O (5 min, 6,000 × *g*), 1.6 mL 70% (vol%) ethanol was added while vortexing. After overnight incubation, the suspended cells were pelleted (4,000 × *g*, 4 min) and washed once with 500 µL of 50 mM Tris-HCl (pH 7.5), after which the cells were resuspended in 100 µL of 50 mM Tris-HCl (pH 7.5) with 1 mg ∙ mL^−1^ RNAse A and incubated at 37˚C for 2 h. Subsequently, 200 µL of 50 mM Tris-HCl (pH 7.5) + 7.5 mg∙ mL^−1^ trypsin was added, and the solution was vortexed and incubated at 37˚C for 2 h. Afterward, the cells were centrifuged and washed once with 200 µL of 50 mM Tris-HCl (pH 7.5) and resuspended in 500 µL of 50 mM Tris-HCl (pH 7.5). Per sample, 1 mL of 50 mM Tris-HCl (pH 7.5) + 1 µM Sytox Green was prepared in dark Eppendorf tubes, and 100 µL of the sample was added, vortexed, and stored on ice. Cells were sonicated at 30% (6 µm) for 15 s using a Soniprep 150 (Medical and Scientific Equipment, Nuaillé, France). An Accuri C6 flow cytometer (Becton Dickinson, Franklin Lakes, NJ) was used to derive the distribution of DNA content per sample.

### Microscopy

Strains were grown to stationary phase in SM supplemented with the appropriate carbon source and imaged with a Zeiss light microscope equipped with an AxioCam MR3 camera (Carl Zeiss, Jena, Germany) in bright-field mode using ZEN software (provided by the device manufacturer).

### Sedimentation tests

Sedimentation was visualized and quantified as described previously (21). In short, yeast cells were harvested from fully grown shake-flask cultures on SM with their corresponding carbon source, washed, and resuspended in SM to a biomass concentration of 2 g dry weight ∙ L^−1^. After homogenizing the suspension by vortexing, they were placed in test tubes and imaged after 5 and 10 min.

To quantify the sedimentation rate, biomass from fully grown shake-flask cultures was washed and resuspended in SM to a concentration of 0.42 g dry weight ∙ L^1^. The suspension was incubated in a 1.5 mL cuvette for 30 min, while the OD_660_ was continuously recorded using a Jenway 7200 Spectrophotometer (Bibby Scientific, Staffordshire, UK).

To simulate biomass retention inside a sequential batch reactor at a smaller scale, biomass from stationary-phase shake-flask cultures grown on SM with their corresponding carbon source was washed and resuspended in SM to a concentration of 2 g dry weight ∙ L^1^. Ten milliliters of this suspension were added to 15 mL tubes and briefly vortexed to ensure homogeneity. Either immediately or after a 30 min settling time, 1 mL samples were carefully drawn at 1 min intervals from a fixed sampling height at the 1 mL mark of the tube and added to a second tube. The concentration of biomass in the remaining 1 mL was calculated by measuring biomass in the second tube and subtracting it from the total biomass.

### Batch and sequential batch cultivation

Anaerobic batch cultures were grown in 2 L laboratory bioreactors (Applikon, Delft, the Netherlands) with a working volume of 1 L, continuously sparged with 0.5 L N_2_ ∙ min^−1^ and stirred at 400 rpm. Temperature was set to 30˚C, and pH was controlled at 5.0 by automated addition of 2 M KOH. Foaming was reduced by adding 0.2 g ∙ L^−1^ antifoam C (Sigma-Aldrich) to the medium. Precultures were centrifuged and resuspended in sterile demineralized water before inoculation 0.2 g dry weight ∙ L^1^. In the co-culture cultivations, the inoculum was a 1:1 mix of both strains, combined prior to inoculation. Bioreactor exhaust gas was cooled by a condenser to 2˚C, dried with a Perma Pure dryer (Inacom Instruments, Veenendaal, the Netherlands), and CO_2_ concentrations were continuously recorded using an NGA 2000 analyser (Rosemount Analytical, Orrville, OH).

**Fig 6:**
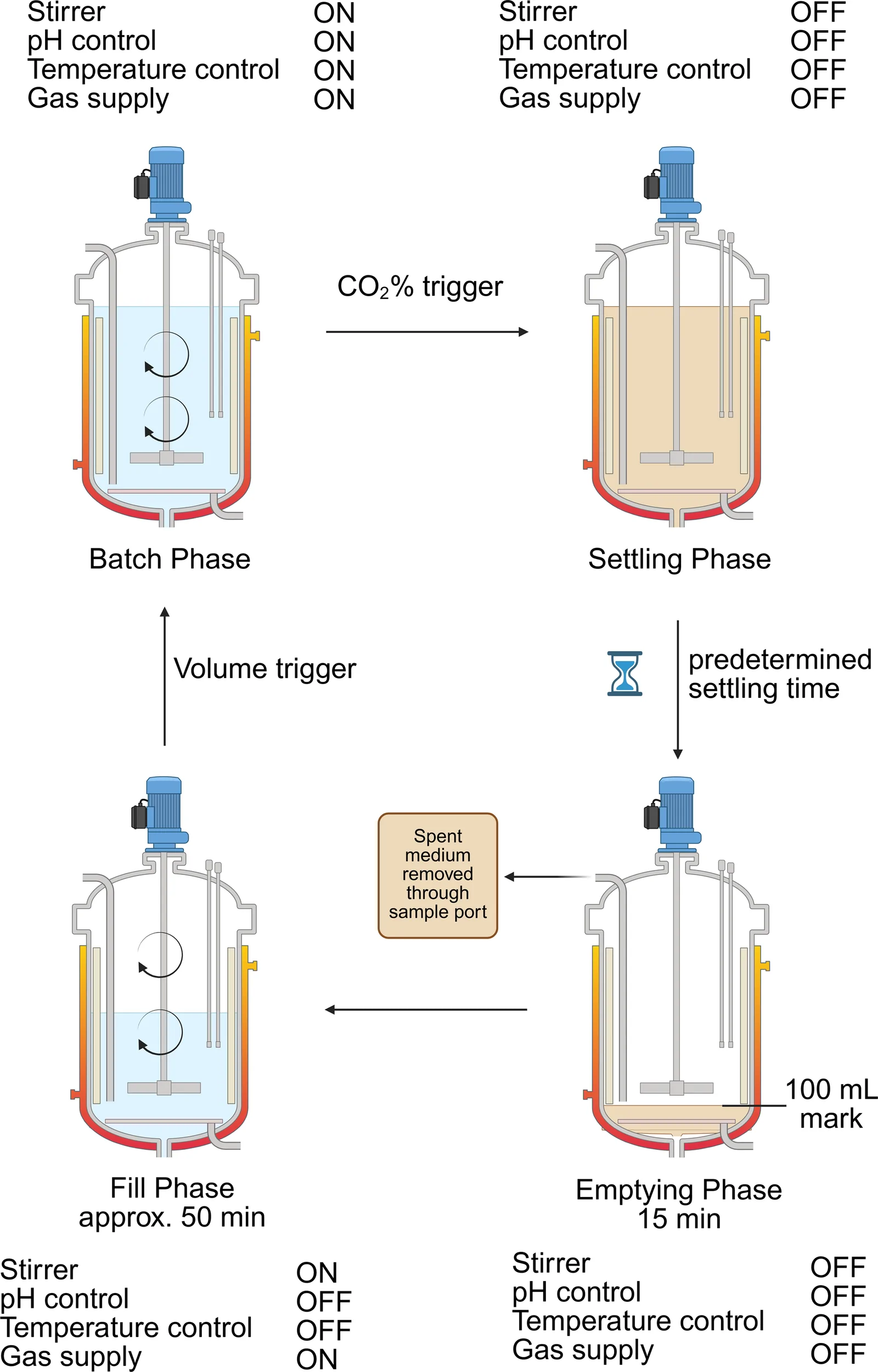
Setup for sequential batch reactors with biomass settling phase. The batch phase, in which the main fermentation occurred, was initiated at a liquid volume of 1 L with gas supply and stirrer, as well as pH and temperature control active. After a drop in CO_2_ % in the off-gas indicated full depletion of the carbon source(s), the settling phase was initiated automatically, and the stirrer and gas supply were turned off. At the same time, pH and temperature control were turned off to prevent overheating or incorrect pH adjustment. After a predetermined settling time (e.g., 30 min), 900 mL spent medium was removed through the sampling port (Emptying phase). Subsequently, 900 mL of fresh medium was added, which required ca. 50 min and was terminated automatically by a volume sensor (Fill phase). Here, the stirrer and gas supply were reactivated. After reaching the working volume of 1 L, temperature and pH control were reactivated to initiate a new cycle. The figure was created in BioRender. Fecker, T. (2026): https://BioRender.com/ds97o4y.

Sequential batch cultivation was set up by programming an automated empty-refill sequence, using the off-gas CO_2_ concentration as a trigger (Fig. 6). In each cultivation cycle (Batch phase), stirrer and gas supply were turned off when the off-gas CO_2_ had reached 2% of the maximum registered CO_2_ concentration during the cycle, indicating that the culture had exhausted the medium and entered stationary phase. The culture was then left to settle for a predetermined time (Settling phase), at which the stirrer and gas supply was turned off, as well as the pH and temperature control. Subsequently, 900 mL of spent medium was automatically removed via the sampling port (Emptying phase, approx. 15 min). The bioreactor was refilled using 900 mL fresh medium with the stirrer and gas supply reactivated (Fill phase, approx. 50 min) until a volume-based trigger was activated. After refilling, the temperature and pH control were reactivated, initiating a new batch phase. The cycle time was defined as the time from the start of the fill phase until reaching the settling phase. The reactor was operated in sequential batch mode for 7 ± 1 cycles.

### Analytical methods

Biomass dry weight was measured gravimetrically by filtering 10 mL samples over pre-weighed nitrocellulose filters (pore size 0.45 µm) in duplicates. Filters were washed with demineralized water and dried in a microwave oven for 20 min at 360 W. Specific growth rates were based on at least five biomass dry weight samples taken during the exponential growth phase.

Metabolite concentrations were determined by high-performance liquid chromatography on an Agilent 1260 Infinity HPLC system (Agilent Technologies, Santa Clara, CA), equipped with a Bio-Rad Aminex HPX 87H ion-exchange column (Bio-Rad, Hercules, CA) eluted at 60˚C with 0.5 mM H_2_SO_4_ as mobile phase at a flow rate of 0.6 mL/min. Metabolite concentrations were quantified using an Agilent G1362A refractive-index detector and an Agilent G1314F VWD detector.

## Data Availability

The authors declare that the data supporting the findings of this study are available within the paper and its supplemental material. The collected raw data are available in a publicly accessible repository (data.4tu.nl) under the CC BY-NC-ND 4.0 license, named "Data supporting the publication: Sedimentation accelerates mixed-substrate conversion during sequential batch cultivation of a co-culture of sugar-specialist yeast strains," DOI: 10.4121/e683cbed-98d7-486f-9bd3-60315dacb05b.v1. Genome sequencing data for strain IMS1278 are available under BioProject accession number PRJNA1490322 at the NCBI BioProject database (https://www.ncbi.nlm.nih.gov/bioproject).

## SUPPLEMENTAL MATERIAL

Supplemental material aem.00516-26-s0001.pdf Tables S1 and S2; Fig. S1 to S4.

ASM does not own the copyrights to Supplemental Material that may be linked to, or accessed through, an article. The authors have granted ASM a non-exclusive, world-wide license to publish the Supplemental Material files. Please contact the corresponding author directly for reuse.
