# Supplementary material for: Sedimentation accelerates mixed-substrate conversion during sequential batch cultivation of a co-culture of sugar-specialist yeast strains
Source: Appl Environ Microbiol. 2026 Jul 16;92(8):e00516-26. doi: 10.1128/aem.00516-26 (PMC13488295; doi:10.1128/aem.00516-26)
Supplement: Supplementary file 1 [file aem.00516-26-s0001.pdf]

Supplementary information

Sedimentation accelerates mixed-substrate conversion during sequential batch cultivation of a co-culture of sugar-specialist yeast strains

Tobias Fecker[1], Michelle Rossouw[1], Jack T. Pronk[1], Rinke J. van Tatenhove-Pel[1]

[1] Department of Biotechnology, Delft University of Technology, Van der Maasweg 9, 2629 HZ Delft, The Netherlands

Corresponding author: Rinke van Tatenhove-Pel; e-mail R.J.vanTatenhove-Pel@tudelft.nl

Table S1: Plasmids used in this study.

Plasmid	Characteristics	Reference
pROS13	2 μm ori, ampR, kanMX, gRNA-CAN1.Y, gRNA-ADE2.Y	[1]
pUDR913	2 μm ori, ampR, kanMX, gRNA-ACE2	This work

Table S2: Oligonucleotides used in this study. All oligonucleotides were purified by desalting.

Oligonucleotide name	Sequence
6005	GATCATTTATCTTTCACTGCGGAGAAG
20864	TGCGCATGTTTCGGCGTTCGAAACTTCTCCGCAGTGAAAGATAAATGATCATCCGAGGCGATATTAACGTGTTTTAGAGCTAGAAATAGCAAGTTAAAATAAG
20865	ATCATGCATAGATAAATGTTCGTCAGAGAGCATCAGTTTCGTTTGAAAGGGTGCGGTTCGGTTATCCATGATTGTATTAACACCGTTTTTGGTCCTATAGATTTCTTTAGTTATTTCTTG

20866	CAAGAAATAACTAAAGAAATCTATAGGACCAAAAACGGTG<br>TTAATACAATCATGGATAACCGAACCGCACCCTTTCAAAC<br>GAAACTGATGCTCTCTGACGAACATTTATCTATGCATGAT
20867	GATGCACCGTTGTATTTTCG
20868	CCGTCACTCCATTAGAATCC

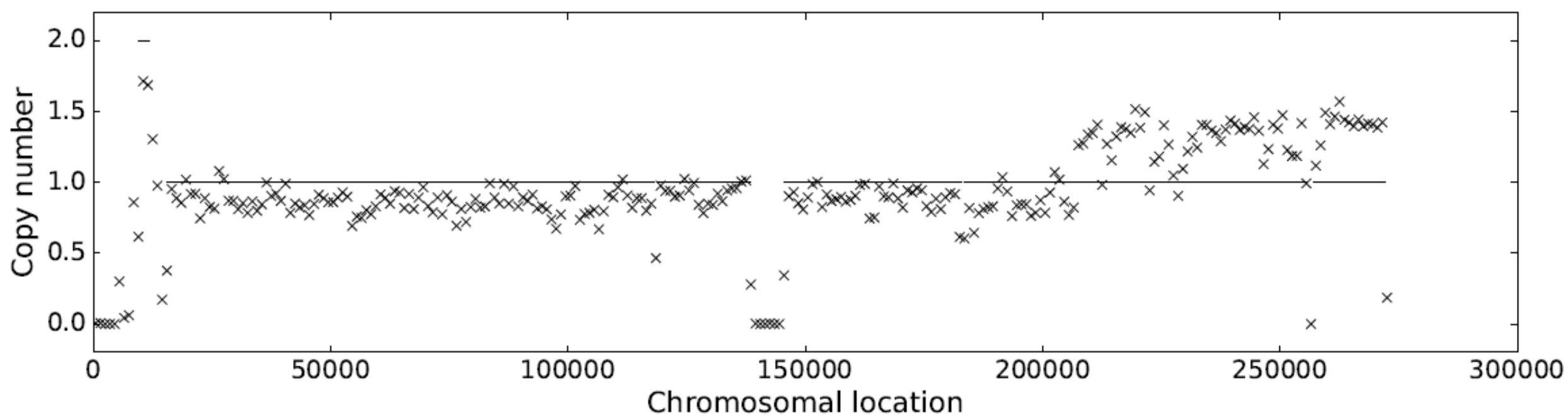

Figure S1: Computational analysis of sequencing coverage using the MAGNOLYA algorithm. As a representative for all chromosomes chromosome VI is shown here. Within the algorithm, the coverage is mapped to a reference representing the average coverage of the whole genome. Partial duplications are indicated with a whole integer copy number (e.g. 2). In the above graph, there is a partial copy number change to ca. 1.5. Since the copy number must be an integer, this suggests that the whole genome had undergone duplication and the region from ca. 200,000 onwards has a copy number of n=3.

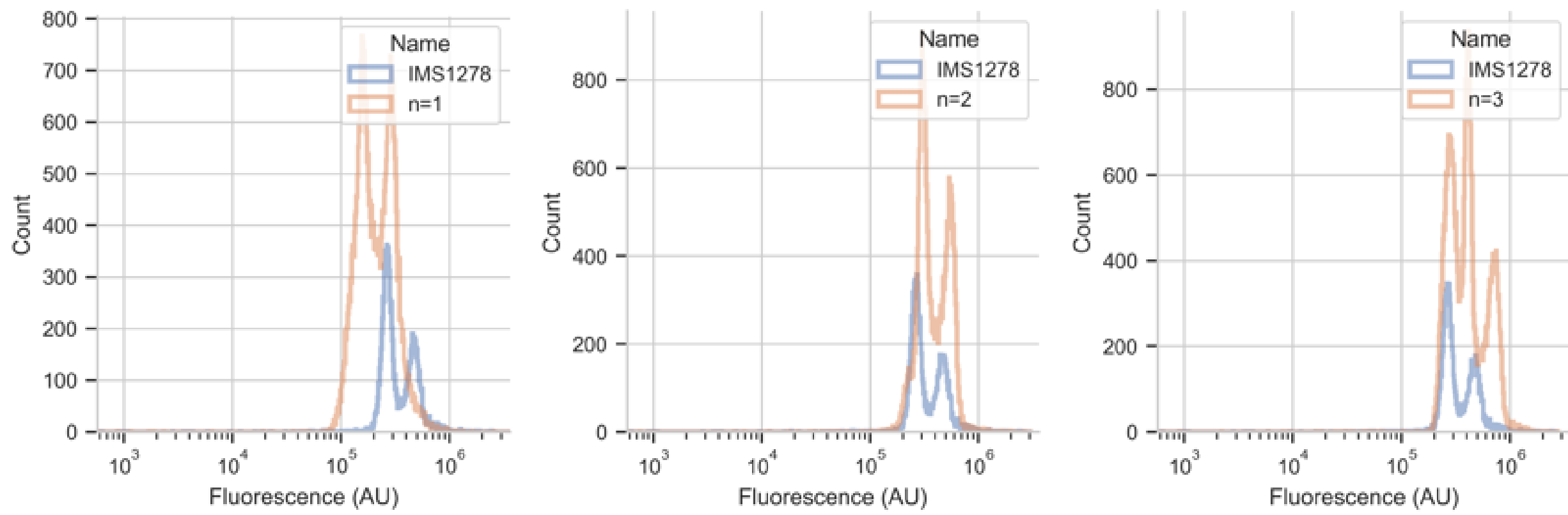

Figure S2: Ploidy test of the isolated strain IMS1278 against reference S. cerevisiae strains with haploid (CEN.PK 113-7D, left panel), diploid (CEN.PK122, middle panel) or triploid (FRY153, right panel) genomes. The strains were stained with Sytox Green and then analysed using flow cytometry. The matching flow cytometry pattern showed that IMS1278 had a diploid genome.

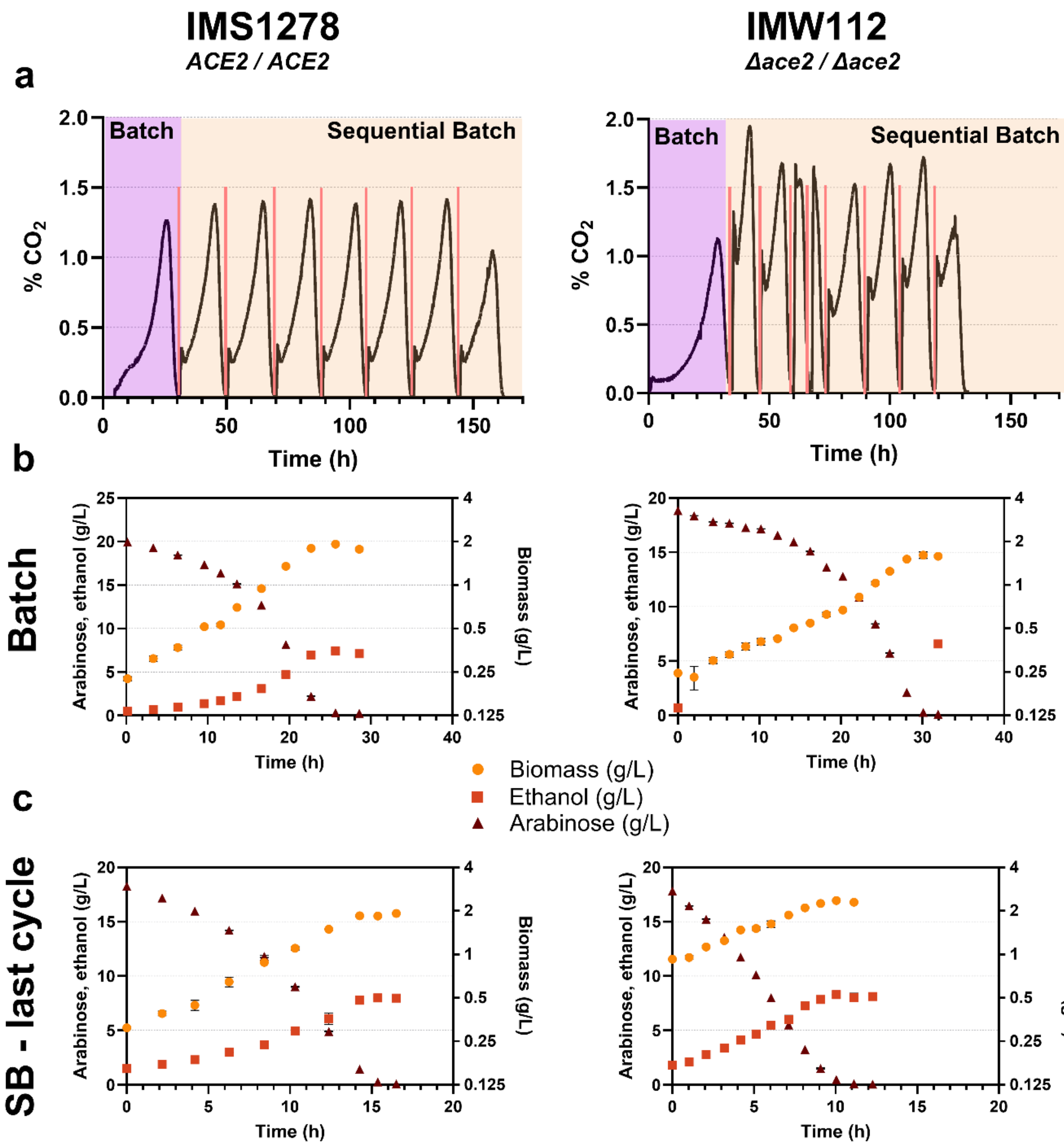

Figure S1: Fermentation kinetics of diploid *S. cerevisiae* strains IMS1278 (*ACE2/ACE2* L-arabinose -specialist strain, left-hand panels; see Table 2 for genotype description) and IMW112 (*Δace2/Δace2* mutant of strain IMS1278, right-hand panel), grown in anaerobic sequential-batch bioreactors on SM with 20 g · L^{-1} L-arabinose. a) CO_2

content of bioreactor off-gas during the initial batch culture and seven subsequent sequential batch cycles. Off-gas data for the final cycle are influenced by sampling for metabolite analyses. The 30-min settling phase is indicated in red. b) Concentrations of arabinose, biomass and ethanol in the initial anaerobic batch cultures of each strain. Cultures were inoculated with 0.2 $g{\cdot}L^{-1}$ biomass. Due to an issue with the ethanol sampling procedure, only start and endpoint ethanol concentrations could be determined for the IMW112 strain c) Concentrations of arabinose, biomass and ethanol in the seventh cycle of sequential-batch operation of each strain. Metabolite and biomass data shown in the figure represent average ± SD of technical replicates. Duplicate bioreactors were run for each strain, with data from the second one shown in the figure.

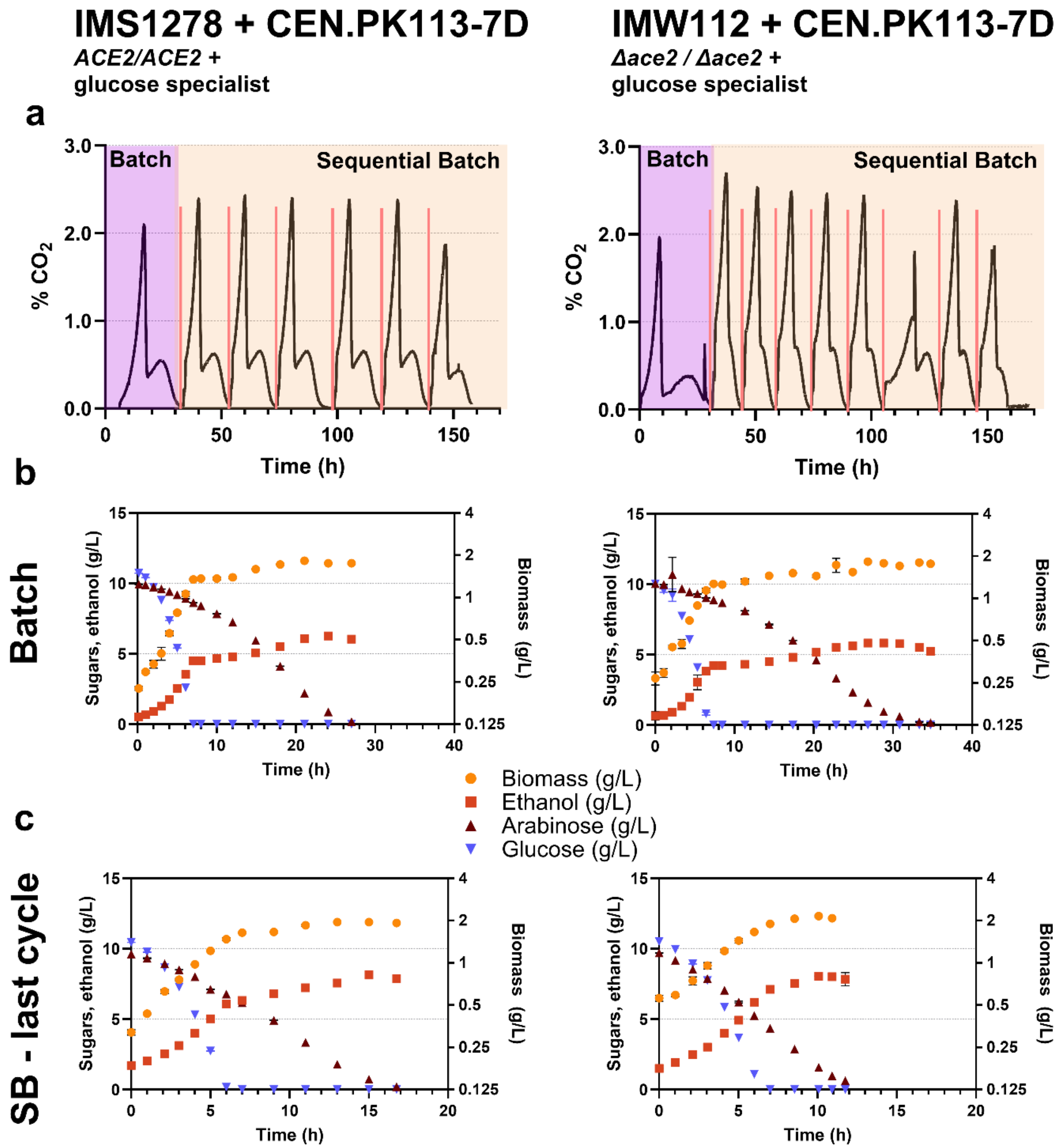

Figure S3: Fermentation kinetics of co-cultures of the pentose-non-fermenting reference strain ('glucose specialist') *S. cerevisiae* CEN.PK with either strain IMS1278 (*ACE2/ACE2* arabinose -specialist strain, left-hand panels; see Table 2 for genotype description) or IMW112 *(*Δ*ace2/*Δ*ace2* mutant of strain IMS1278, right-hand panels), grown in anaerobic sequential-batch bioreactors on SM with 10 g · L^{-1} each of glucose

and arabinose. A. CO_2 content of bioreactor off-gas during and initial batch culture and subsequent sequential-batch cycles. Off-gas data for the final cycles are influenced by sampling for metabolite analyses. The 30-min settling phase is indicated in red. B. Concentrations of L-arabinose, biomass and ethanol in the initial anaerobic batch cultures of each co-culture, inoculated with 0.1 g biomass·L^{-1} of each strain. C. Concentrations of arabinose, glucose, biomass and ethanol in the final cycle of sequential-batch operation. Metabolite and biomass data shown in the figure represent average ± SD of technical replicates. Duplicate bioreactors were run for each strain, with data from the second one shown in the figure.

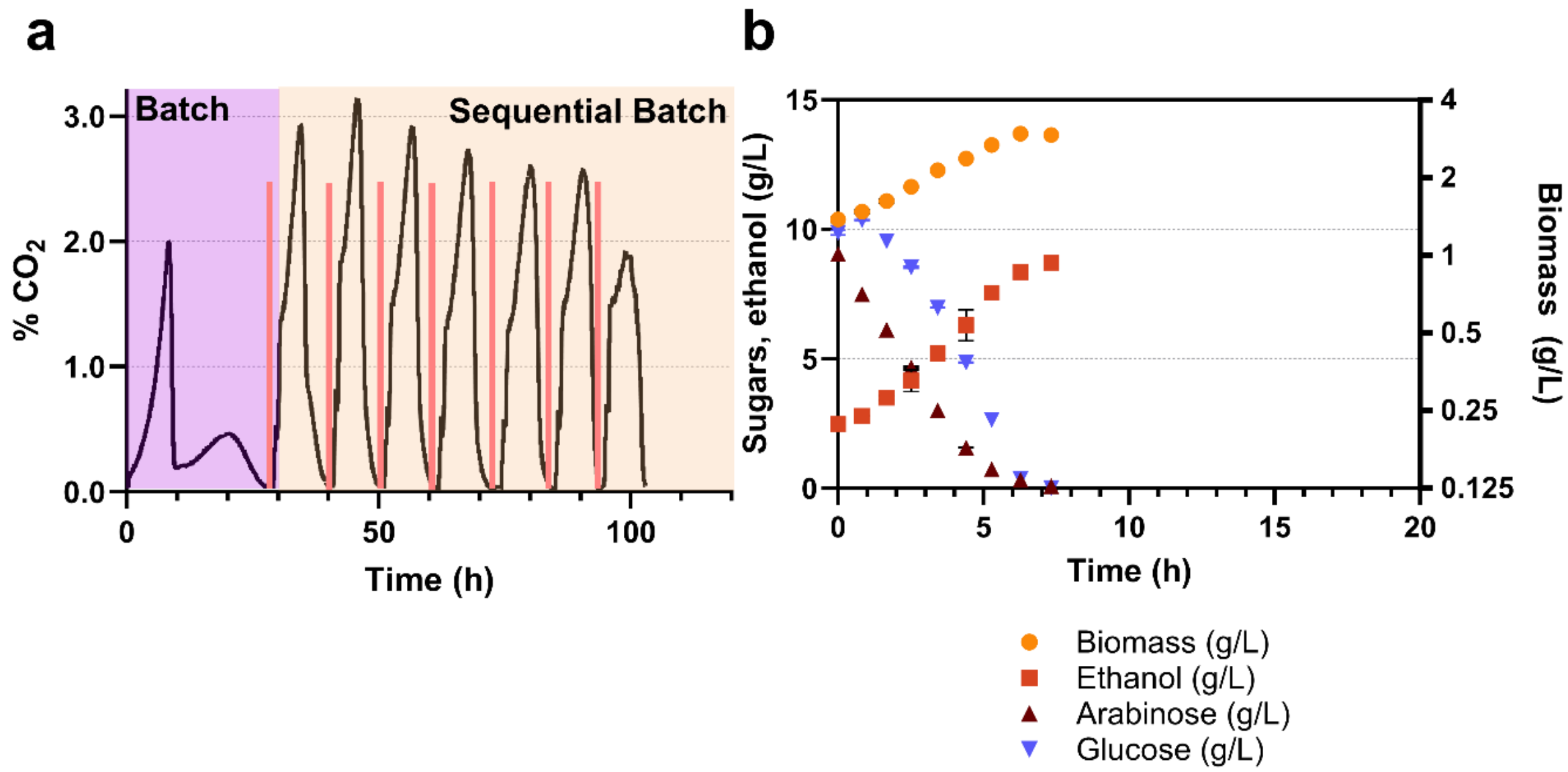

Figure S4: Fermentation kinetics of co-cultures of the pentose-non-fermenting reference strain (‘glucose specialist’) *S. cerevisiae* CEN.PK and the arabinose - specialist strain IMW112 *(Δace2/Δace2*) grown in anaerobic sequential-batch bioreactors on SM with 10 g · L^{-1} each of glucose and arabinose. In comparison with the experiments shown in Figure 4, settling time after substrate depletion was extended from 30 to 60 min. a) CO_2 content of bioreactor off-gas during and initial batch culture and subsequent sequential-batch cycles. Off-gas data for the final cycle are influenced by sampling for metabolite analyses. The 60-min settling phase is indicated in red. b) Concentrations of arabinose, glucose, biomass, and ethanol in the final cycle of sequential-batch operation. Metabolite and biomass data shown in the figure represent average ± SD of technical replicates. Duplicate bioreactors were run for each strain, with data from the second one shown in the figure.

Bibliography

[1] R. Mans *et al.*, “CRISPR/Cas9: A molecular Swiss army knife for simultaneous introduction of multiple genetic modifications in Saccharomyces cerevisiae,” *FEMS Yeast Res.*, vol. 15, no. 2, pp. 1–15, 2015, doi: 10.1093/femsyr/fov004.
